# Development and Evaluation of Oromucosal Spray Formulation Containing Plant-Derived Compounds for the Treatment of Infectious and Inflammatory Diseases of the Oral Cavity

**DOI:** 10.3390/polym16182649

**Published:** 2024-09-19

**Authors:** Yuliia Maslii, Nataliia Herbina, Lina Dene, Liudas Ivanauskas, Jurga Bernatoniene

**Affiliations:** 1 Department of Drug Technology and Social Pharmacy, Lithuanian University of Health Sciences, LT-50161 Kaunas, Lithuania; yuliia.maslii@lsmu.lt (Y.M.); nataliia.herbina@lsmu.lt (N.H.); 2 Department of Industrial Technology of Drugs, National University of Pharmacy, 61002 Kharkiv, Ukraine; 3 Laboratory of Biochemistry and Technology, Institute of Horticulture, Lithuanian Research Centre for Agriculture and Forestry, LT-54333 Babtai, Lithuania; lina.dene@lammc.lt; 4 PetalNord MB, Kruosto g. 31, LT-47214 Kaunas, Lithuania; 5 Department of Analytical and Toxicological Chemistry, Lithuanian University of Health Sciences, LT-50161 Kaunas, Lithuania; liudas.ivanauskas@lsmu.lt; 6 Institute of Pharmaceutical Technologies, Faculty of Pharmacy, Medical Academy, Lithuanian University of Health Sciences, LT-50161 Kaunas, Lithuania

**Keywords:** oromucosal spray, clove CO_2_ extract, essential oils of lavender and grapefruit, emulsion, mucoadhesive polymers, development, evaluation

## Abstract

According to data in the literature, natural products and essential oils are often used in dental practice. To develop a new oromucosal spray for the treatment of infectious and inflammatory diseases of the oral cavity, clove CO_2_ extract and essential oils of lavender and grapefruit were used as active pharmaceutical ingredients. Clove extract was obtained by the method of subcritical extraction from various raw materials, the choice of which was based on the yield of the CO_2_ extract and the study of its phytochemical and microbiological properties. Based on the results of microscopic and diffraction analyses, the rational time of ultrasonic exposure for the emulsion of active pharmaceutical ingredients was established. Mucoadhesive polymers were used as stabilizers of the two-phase system and prolongators. This article discusses the impact of the type and concentration of mucoadhesive polymers on the stability of the emulsion system; the viscous, textural, adhesive, and film characteristics of oromucosal spray; and the parameters determining sprayability.

## 1. Introduction

The problem of infectious and inflammatory diseases of the oral cavity (periodontitis, gingivitis, stomatitis, etc.) is one of the most urgent and widespread in dental practice [1]. These pathologies often lead to the loss of teeth and the appearance of foci of chronic infection, and as a result, a decrease in the reactivity of the body as a whole [2,3,4].

The main symptoms accompanying infectious and inflammatory diseases of the oral cavity include irritation, inflammation, redness, and bleeding of the gingival tissue, pain, changes in the oral microbiota, the formation of ulcers, bad breath, purulent discharge in severe cases, etc. [5,6].

Despite significant successes in the creation of chemical pharmaceuticals, the popularity of phytotherapy in dentistry has increased in recent years. This is due to the unique properties of medicinal plants, which contain many biologically active substances and are characterized by a wide spectrum of pharmacological action. In addition to their highly effective complex action, the absolute advantages of herbal preparations include a mild therapeutic effect that develops gradually, a much smaller number or even the absence of contraindications and side effects, low toxicity, the inability to cause addiction, and the possibility of long-term use in different age categories [7,8,9].

Clove (*Syzygium aromaticum* L.) is an affordable and effective natural remedy to treat various oral diseases [10,11,12]. A number of publications reveal the effectiveness and functional properties of both clove oil and extract [13,14,15,16]. Due to its rich and diversified composition of biologically active compounds, clove has anti-inflammatory, antibacterial, antifungal, and analgesic properties that can effectively reduce inflammation caused by a variety of periodontal and mucous membrane diseases, provide relief from toothache pain, and solve other dental problems caused by bacteria and infections [17,18,19]. Eugenol, the main bioactive compound in cloves, is responsible for their powerful antioxidant, analgesic (pain-relieving) and antiseptic properties [20,21,22]. Moreover, cloves can help to get rid of the problem of bad breath [23]. Subcritical CO_2_ extraction is a green, low-temperature method that preserves thermolabile compounds and is suitable for the extraction of clove [24]. However, we identified a lack of information about CO_2_ extract applications in dental formulations.

The major active components of lavender oil are linalool and linalyl acetate, the activity of which reflect that of the whole oil [25,26]. It exhibits good antimicrobial and antifungal activities against most bacteria, filamentous fungi, and yeasts [27,28]. Pathan J.M. and co-authors proved that lavender oil can be an effective local analgesic agent and a good alternative to chemical anaesthetics [29]. Citrus essential oils, including grapefruit, are also widely used in the food, cosmetic, and pharmaceutical industries [30,31]. Like most citrus essential oils, the main components of grapefruit oil are terpenes and terpene oxides, which demonstrate antifungal, antiviral, and anti-inflammatory properties, making them versatile remedies for oral health care [32,33]. Studies of other researchers have shown the substantial antibacterial activity of grapefruit essential oil against *Bacillus cereus, Enterococcus faecalis, Escherichia coli, Klebsiella pneumonia, Pseudococcus* sp., *Salmonella thyphimurium*, *Shigella flexneri*, and *Staphylococcus aureus*, as well as a strong antifungal activity against *Aspergillus Niger, Candida albicans, Cladosporium cucumerinum, Penicillium digitatum, Penicillium italicum,* and *Penicillium chrysogenum* [34,35]. The study by Gugulethu Miya and co-authors reveals that the essential oils from grapefruit are non-toxic and demonstrate significant anti-inflammatory activity [36]. Deng W. and co-authors established the antimicrobial, antioxidant, and antiproliferative properties of grapefruit essential oil [37].

Based on such data, we chose to investigate combinations of clove CO_2_ extract and lavender and grapefruit essential oils as our research object. The expected outcome of this combination is the ability to effectively contribute to the prevention and treatment of infectious and inflammatory processes, namely, to inhibit the activity of pathogenic microflora in the oral cavity, provide anti-inflammatory and analgesic effects, eliminate unpleasant odours, stimulate reparative processes, and epithelize affected areas of the periodontium and mucous membrane.

Considering the etiopathogenesis of the infectious and inflammatory diseases of the oral cavity, their treatment mostly requires the use of local drugs. The local treatment of periodontal and mucous membrane diseases has significant interest, owing to benefits such as minimal systemic side effects and higher local drug concentrations [38].

Oromucosal sprays are one of the most popular and widespread forms of local drug administration for infectious and inflammatory processes in the oral cavity. Their main difference from other dosage forms is the presence of a dosing pump, which creates an air-drop jet via mechanical pressure on the dosing pump. This dosage form has several advantages: increased pharmacological activity due to the dispersion of the medicinal substance, a high degree of exposure to mouth mucous membranes, rapid absorption, the exclusion of the negative effect of the digestive tract on the active substance, tightness, dosage accuracy, painlessness, convenience, and speed of use, as well as simplicity and economy of production [39,40,41].

However, there are also disadvantages, such as low adhesion to the surface of mucous membranes, which is associated with the constant formation of saliva and the mobility of oral cavity tissues; a rapid decrease in concentration due to the dilution and washing of medicinal substances with saliva; their low bioavailability; and the need for frequent use. Therefore, to increase the retention time of the spray dosage form on mucous membranes and ensure a prolonged effect, mucoadhesive polymer compounds are introduced into its composition, which play the role of film and structure formers, stabilizers, and fillers [42,43,44,45]. Wetting mucoadhesive polymers results in the formation of a viscous solution that prolongs adherence to the mucosal surface. This, in turn, causes more adhesive interactions, such as the forming of hydrogen bonds, electrostatic interactions, and covalent bonding, increasing the bioavailability of the active pharmaceutical ingredient (API) by extending the time the dosage form spends at the site of absorption [46,47]. Mucoadhesive polymers must meet certain conditions; namely, they must be allowed for daily use, provide the long-term release of drugs, adhere quickly, and not irritate the mucous membrane of the oral cavity [48].

Typically, when choosing a mucoadhesive, the following physicochemical properties are taken into account: molecular weight (usually should be in the range from 10 to 4000 kDa to allow polymers to hydrate easily, as well as release active groups for interaction with mucin and at the same time form strong gels); polymer chain length (to facilitate the interpenetration of macromolecules and not interfere with diffusion); molecular conformation (to ensure accessibility for chemical interactions with mucin); and the flexibility of polymer chains (to ensure interpenetration, the adhesion of polymer molecules with mucin and, accordingly, the controlled release of the drug) [47,49].

Mucoadhesive polymers in oral drug delivery systems may be natural (sodium alginate, chitosan, pectin, gelatine, agarose, guar and xanthan gum, hyaluronic acid, etc.) [45,50,51,52], semi-synthetic (cellulose derivatives: hydroxyethyl cellulose (HEC), hydroxypropyl cellulose (HPC), sodium carboxymethylcellulose (sodium CMC), hydroxypropyl methyl cellulose (HPMC), methyl cellulose, etc.) [46,53,54], or of synthetic origin (polyethylene glycols, poloxamers, polylactides, polyamides, acrylic acid polymers, polyvinyl alcohol (PVA), polyvinylpyrrolidone (PVP), etc.) [42,55,56,57].

The above biopolymers, especially polysaccharides and their derivatives, such as chitosan, hyaluronic acid, alginate, carrageenan, cellulose derivatives, etc., are most often used as mucoadhesive carriers in the development of drugs for dental practice. They are generally stable, nontoxic, hydrophilic, and biodegradable, and display reactive functional groups (e.g., carboxyl or amino groups) that promote mucoadhesion. Their properties can be modified to improve certain qualities, including solubility, viscosity, and stability [58,59].

The use of polymers as drug carriers is constantly growing among mucoadhesive oral forms. In the work of Madrazo-Jiménez et al., the benefit of chitosan gel in the healing of surgical wounds of the oral cavity was noted [60]. A polymeric film for the buccal delivery of the antifungal drug posaconazole based on alginate and its oligosaccharide oligomer as a mucoadhesive biopolymer, developed by Szekalska et al. [61], and a buccal mucoadhesive film with cetirizine based on the biopolymers sodium alginate and HPMC, prepared by Pamlény et al. [62], were shown to have high mucoadhesive and swelling properties. Elbanna et al. produced mucoadhesive composite sponges based on κ-carrageenan and xanthan biopolymers for the buccal delivery of tetrahydrocurcumin for the prevention and treatment of oral cancer, demonstrating good mucoadhesion properties and prolonged drug release [63]. Tonglairoum et al. developed multilayer oromucosal patches to improve the topical therapy of oral lichen planus and recurrent aphthous stomatitis based on l-cysteine-thiolated chitosan, 2-hydroxypropyl-β-cyclodextrin, and PVP [64].

However, the use of mucoadhesive polymers in the development of oral sprays containing compounds of plant origin, where they act as a stabilizer of two-phase systems, is insufficiently covered.

Therefore, the aim of our study is the development and evaluation of oromucosal spray formulation containing plant-derived compounds for the treatment of infectious and inflammatory diseases of the oral cavity.

## 2. Materials and Methods

### 2.1. Materials

The list of ingredients, their manufacturers, and role that they play in the oromucosal spray are given in Table 1.

The following emulsifiers approved for oral use were used: L-α-Lecitin soybean (Sigma-Aldrich, St Saint Louis, CA, USA) (a concentrate of soybean lecithin consisting of more than 94% phosphatidylcholine and less than 2% triglycerides), molecular weight—758.075 g/mol; polysorbate 80 (Carl Roth GmbH, Roth, Germany), 97% purity, molecular weight—1310 g/mol; sodium caseinate (Sigma-Aldrich, Steinheim, Germany), 98% purity, molecular weight—314.440 g/mol; sucrose stearate Sisterna^®^ SP70-C (Sisterna, Roosendaal, The Netherlands), purity (ester content) above 80%, molecular weight—608.758 g/mol.

A dark glass 50 mL bottle with a mechanical pump-type sprayer was chosen for the packaging of the spray being developed, allowing the drug to be finely sprayed and, accordingly, cover a large surface area of the mucous membrane.

### 2.2. Clove CO_2_ Extract Preparation

Different types of *Syzygium aromaticum* L. materials were used for extraction: (1) whole buds, (2) stems, and (3) bud powder. These raw materials were purchased from PW Rekord Józef Szwajkowski (Zelazkow, Poland). Whole buds and stems were ground to homogenic powder using an RRH-2000A electric grinder mill (Zhejiang Yongtian Mechanical and Electrical Manufactured Co., Ltd., Yongkang, Zhejiang, China) before the extraction. In total, 10 kg of each material was used for the subcritical CO_2_ extraction process, as described by Šernaitė et al. [65], with some parameter modifications: T = 10 °C, P = 40 bar, t = 16 h. Collected extracts were filtered and stored at 4 °C until further experiments.

The obtained clove CO_2_ extracts were weighted using scales, and each extract yield was calculated using Formula (1):

Yield (%) = (mass of the extract, kg/mass of the raw material, kg) × 100(1)

### 2.3. Preparation of API Emulsion

Ultrasound-assisted emulsification was performed to obtain the API emulsions (o/w). A UP400St Ultrasonic Processor (400 W, 24 kHz) (Hielscher Ultrasonics GmbH, Teltow, Germany) was used to prepare emulsions from the clove CO_2_ extracts, obtained previously, with combinations of essential oils of lavender and grapefruit. The emulsions of the API combinations and water were prepared at different emulsification durations: 5, 10, 20, 30, 40, 50, and 60 min. An ice bath was used to control the temperature to not exceed 40 °C.

### 2.4. Oromucosal Spray Preparation

Sodium benzoate, potassium sorbate, sucralose, and grapefruit powder flavour were dissolved in the required amount of purified water using a magnetic stirrer with a stirring speed of 400–500 rpm. A mucoadhesive polymer was added to the resulting solution (sodium alginate, Na-CMC, and HPC were dissolved at room temperature; PVA was heated to a temperature of 80–90 °C; HEC, xanthan, PVP—50–60 °C), mixed, and left until complete swelling and the formation of a low-viscosity homogeneous solution had taken place. In a separate container, essential oils of lavender and grapefruit were dissolved in ethyl alcohol 96%; clove CO_2_ extract was added and thoroughly mixed with the necessary amount of water, using US exposure. The resulting API emulsion was added into a solution with mucoadhesive (pre-cooled to a temperature no higher than 30–40 °C to avoid the evaporation of essential oils) and thoroughly mixed with an Ultra-Turrax IKA T18 homogenizer (Staufen, Germany) at 10,000 rpm for 3–5 min until a homogeneous system was formed.

### 2.5. Research Methods

#### 2.5.1. Film-Forming Ability

**Figure 1:**
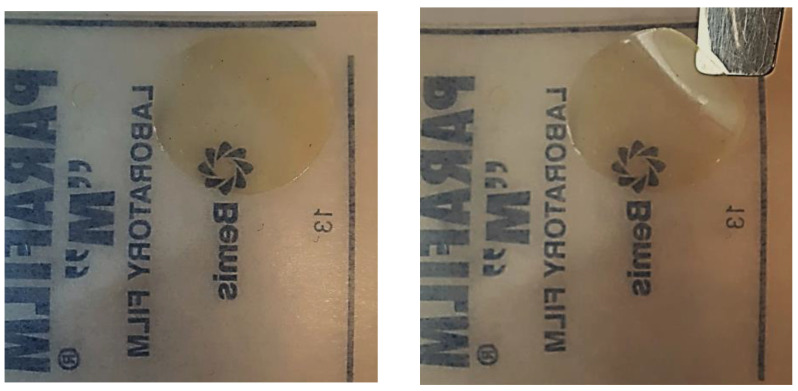
Film-formation test of sprays.

Test samples (1.0 g) were sprayed onto moisture-resistant elastic films (“Parafilm”) and dried at a temperature of (37 ± 0.2) °C. After this, the quality of the resulting films was visually assessed (Figure 1).

#### 2.5.2. Determination of Spray Stability

Test samples (1.0 ± 0.5 g) were placed in special centrifuge tubes (Eppendorf, Hamburg, Germany) and centrifuged in a centrifuge (high-speed centrifuge: Sigma 3-18KS, Berlin, Germany). The test was performed at a temperature of (25 ± 2) °C and speed/time rates of 3000 rpm/5 min and 10,000 rpm/10 min. The stability of the system was assessed by visual observation of the separation of the oil and water phases.

#### 2.5.3. Determination of pH Value

pH level was determined potentiometrically using a pH meter Thermo Scientific Orion VERSA STAR^TM^ Advanced Electrochemistry Meter (Beverly, MA, USA). Measurements were carried out at room temperature and repeated three times.

#### 2.5.4. Viscometric Evaluation

The viscosity of the samples was measured using a Fungilab Alpha series rotational viscometer (Fungilab, Barcelona, Spain). Equal amounts (50.00 ± 0.01 g) of the samples were analysed with an L2/L1 spindle at a shear speed of 50 rpm. Three replicate analyses were performed at room temperature for each sample.

#### 2.5.5. Microscopic Analysis

The particles of the oil phase of the produced samples were observed using a Nikon DS-Fi1 (Tokyo, Japan) optical microscope, the camera of which was connected to a computer. The images were magnified 100 times. For each sample, 10 random droplets of oil were selected, and their average diameter was determined.

#### 2.5.6. Particle Size and Distribution Measurements

Oil particle size and distribution were assessed using a Mastersizer 3000 with a Hydro EV unit (Malvern Panalytical Ltd., Malvern, UK). Samples of emulsions and sprays without dilution were added dropwise in the dispersant (water) to obtain laser obscuration between 9.5% and 10.5%. The pump speed was kept constant at 2400 rpm. The refractive index used for the dispersant was 1.330, for the dispersing material was 1.533 (for clove CO_2_ extract and API emulsion), and for the sprays was 1.500. Particle size distribution was measured in five runs and the average was calculated. The formulations were described by percentile (D10, D50, and D90) values.

#### 2.5.7. Textural Analysis

**Figure 2:**
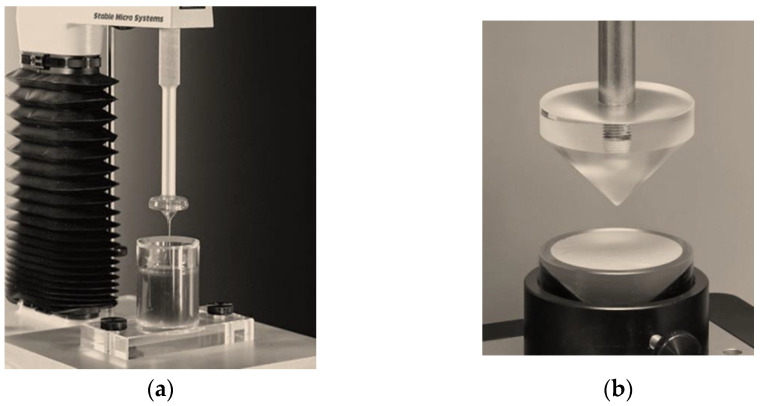
Installation for testing the textural characteristics of the spray on the TA device. XT Plus: (**a**) Back extrusion; (**b**) spreadability.

Tests were performed on a Texture Analyzer (Stable Micro Systems Ltd., Surrey, UK) using an A/BE probe (Figure 2a) for the back extrusion test and an HDP/SR probe for the spreadability test (Figure 2b). Three replicate analyses were performed at room temperature for each sample, ensuring identical conditions for each measurement.

##### Back Extrusion Test

Approximately 50 g of spray was placed in a standard 100 mL sample container, avoiding air and ensuring a smooth surface. A 40 mm diameter disk, which was placed above the surface of the sample at the beginning of the test, was immersed in the spray, resulting in the product being squeezed upward between the walls of the container and the edges of the disk. Study parameters were selected: movement speed (2 mm/s) and immersion depth (10 mm). Spray parameters, such as firmness (maximum compressive force) and consistency (cohesiveness and adhesiveness), were determined from the resulting “force–time” graph. When the piston with the disk moves downward, a positive part of the back extrusion graph is created; the maximum compression force required to deform the spray demonstrates the firmness of the spray mould, and the area of the graph above zero indicates the cohesion of the spray. The higher the value, the denser (more viscous) the consistency of the sample. After the disk returns to its original position, its upward movement creates a negative part of the graph: the area below zero gives an indication of the adhesion and resistance of the sample when pulled away from the disk (minimum retraction force). The higher the value, the more energy is required to break the contact of the sample with the surface of the disk, and, accordingly, the better the adhesive ability of the spray.

##### The Spreadability Test

A spray sample was placed in a cone-shaped receptacle, avoiding air and ensuring a smooth surface. The probe, also cone-shaped, was placed above the surface of the spray at the beginning of the test. Study parameters were chosen: movement speed—2 mm/s; depth of probe immersion in the spray—1 mm. Spray parameters such as firmness, spreadability, and adhesiveness were determined from the resulting “force–time” graph. As the probe moves downward, the spray flows out between the surfaces of the receiver cone and the probe at an angle of 45°. The ease of this process indicates the degree of spreading of the spray; that is, it characterizes its spreadability (area above zero). The peak on the positive side of the graph shows firmness (the ability of the spray to flow). Removing the cone probe from the sample (upward motion) provides information about the adhesive ability of the spray. The value of the adhesion force (separation of the sample from the probe surface) is shown by the maximum peak on the negative part of the graph.

#### 2.5.8. The Geometry of the Spray Plume Study

The geometry of the spray plume is characterized by the spray angle, the structure of the spray plume, and its imprint. The spray angle was defined as the angle of the conical film of liquid at the exit of the bottle until it disintegrated into droplets. The imprint of the plume was determined visually by the area of the irrigated surface, as well as the uniformity and dispersion of the spray droplets. For this, each of the test samples was sprayed once onto the substrate from the same distance (20 cm), and the results were recorded using a photo camera. All photos were taken under the same lighting conditions. The spray was released by manual pressure after a false start to the side, similar to how it is released by the patient. Paper filters were used as the substrate.

#### 2.5.9. Determination of Antimicrobial Activity

The antimicrobial activity of the samples was determined by the diffusion method on Mueller–Hinton agar medium (Mueller-Hinton II Agar, BBL, Cockeysville, MD, USA), using the reference microorganism cultures *Staphylococcus aureus* ATCC 25923, *Staphylococcus epidermidis* ATCC 12228, *Streptococcus mutans* ATCC 25175, *Streptococcus mitis* ATCC 6249, *Enterococcus faecalis* ATCC 29212, *Escherichia coli* ATCC 25922, *Klebsiella pneumoniae* ATCC 13883, *Pseudomonas aeruginosa* ATCC 27853, *Proteus vulgaris* ATCC 8427, *Bacillus cereus* ATCC 11778, and *Candida albicans* ATCC 10231 (all bacteria were obtained from the American Type Culture Collection).

For the assay, nonspored bacteria were grown for 20–24 h at 35–37 °C on BBL™ Trypticase™ Soy Agar medium (ThermoFisher Scientific, Waltham, MA, USA). The bacterial suspension was prepared from cultivated bacterial cultures in a sterile physiological sodium chloride (0.9%) solution and standardized with McFarland’s standard indicator. The bacterial suspension was considered standardized when the indicator value was 0.5 (1 mL of bacterial suspension contains 1.5 × 10^8^ cells of the microorganism).

A reference culture of *Bacillus cereus* spores was grown for 7 days at 35–37 °C on BBL™ Trypticase™ Soy Agar medium. After growing the culture of spore bacteria, it was washed off the surface of the agar medium with a sterile physiological solution. The prepared suspension was heated for 30 min at 70 °C and diluted with saline until the spore concentration per ml was between 10 × 10^6^ and 100 × 10^6^.

A standard fungal culture of *Candida albicans* was grown for 20–24 h at 30 °C on Sabouraud Dextrose Agar medium (ThermoFisher Scientific, Waltham, MA, USA) for 72 h. A fungal suspension was prepared from cultured fungal cultures in physiological saline and standardized with McFarland’s standard indicator.

A 0.5 McFarland turbidity suspension of the standard bacteria was prepared. The bottom of the Petri dishes was divided into 9 segments. The analysis of the reference microorganisms on Mueller–Hinton agar was used to determine the antimicrobial activity of the CO_2_ extracts.

The antimicrobial activity of the spray was determined with the disk-to-agar diffusion method using the reference microorganism cultures on Mueller–Hinton agar medium (Mueller-Hinton II Agar, BBL, Cockeysville, MD, USA). The microbiological analysis of the spray was performed using pure and mixed microbial cultures. For polymicrobial analysis, a bacterial combination of reference microorganisms was prepared: *S. aureus + S. epidermidis + E. faecalis + S. mutans + S. mitis + E. coli.*

##### Determination of CO_2_ Extract Antimicrobial Activity

The chosen amount of CO_2_ extract was poured into a sterile Petri dish (0.2 to 0.005 mL nutrient agar per 1 mL). Then, the necessary quantity of Mueller–Hinton agar to reach 5 mL was added. After the solidification of the agar, suspensions of reference microorganisms were inoculated. Samples were kept in a thermostat for 20–24 h at 35 °C, then stored for 24 h at room temperature. Antimicrobial activity was evaluated. If the cultures had grown, the sample did not inhibit the growth of bacteria. If the reference culture did not grow, the sample had an antimicrobial effect against the microorganism.

##### Determination of Spray Antimicrobial Activity

An amount of 15.0 mL of liquid Muller–Hinton agar at a temperature of 45 °C was poured into nine sterile Petri dishes with a diameter of 90 mm. After the agar solidified, reference cultures of microorganisms were spread on the agar surface of each Petri dish with a sterile swab soaked in the reference suspension of microorganisms using the surface-spread method. After that, sterile disks with a diameter of 8 mm were placed on the agar surface of each Petri dish with a reference microorganism culture. Then, 30 µL of a given sample was dropped onto the surface of each disk. Petri dishes were incubated at 35 °C for 20 h. Positive (does not inhibit the growth of reference cultures) and negative (inhibits the growth of reference cultures) controls were performed. The antimicrobial activity of the sample was assessed by the non-growth of the reference culture around the disk and under the disk (the sample has antimicrobial activity), or growth (the sample did not inhibit the growth of microorganisms).

#### 2.5.10. Quantification of APIs by Gas Chromatography with Flame-Ionization Detection Analysis

The clove CO_2_ extract and spray samples were determined using GC/FID analysis.

GC/FID analysis was performed using a GC-2010 Plus Shimadzu with a flame ionization detector and autosampler AOC-20i+s (Shimadzu Technologies, Kyoto, Japan). The separation of analytes was carried out on an Rxi-5 ms (Restek Corporation, Bellefonte, PA, USA) with a capillary column (30 m long, 0.25 mm outer diameter, and 0.25 μm liquid stationary phase thickness). A robotic autosampler and a split/splitless injection port were used.

The injection port temperature was kept at 250 °C until the end of the analysis. The separation of analytes was carried out on an Rxi-5 ms (Restek Corporation, Bellefonte, PA, USA) with a capillary column (30 m long, 0.25 mm outer diameter, and 0.25 μm liquid stationary phase thickness) with a liquid stationary phase (5% diphenyl and 95% polydimethylsiloxane) with helium at a purity of 99.999% as the carrier gas in a constant flow of 1.18 mL/min. The oven temperature was programmed to 80 °C for 1 min, then increased to 170 °C at 8 °C/min and increased to 220 °C at 35 °C/min, and kept for 5 min. The total time was 20 min. The injection volume was 1.0 μL, and the injection mode was split (split ratio: 50).

The main bioactive constituents were eugenol in the clove CO_2_ extract, linalool and linalyl acetate in the lavender essential oil, and limonene in the grapefruit essential oil. Hexane as a solvent was used in a ratio of 1:10.

The positivity of a substance was confirmed when the chromatogram showed a signal with a relative retention time identical to the reference substance analysed at the same time and under the same conditions.

#### 2.5.11. Statistical Analysis

The mean values and standard deviations of the results were calculated using Microsoft Office 365 Excel 2016 (Redmond, WA, USA). The significance of differences was evaluated using Students *t*-test. The differences were statistically significant at *p* < 0.05.

## 3. Results and Discussion

The first stage of our research was the selection of a rational type of raw material to produce CO_2_ extract. For this purpose, clove CO_2_ extracts were obtained, using whole buds (No. 1) and stems (No. 2), which were ground before the extraction, and bud powder (No. 3). The results showed that the type of raw material affects the yield of extracts. Extract No. 1 had the highest yield of 16.64%; the yields of extracts No. 2 and No. 3 were almost 3.5 times lower than the yield of sample No. 1, and did not differ statistically in value (4.59% and 4.89%, respectively).

**Figure 3:**
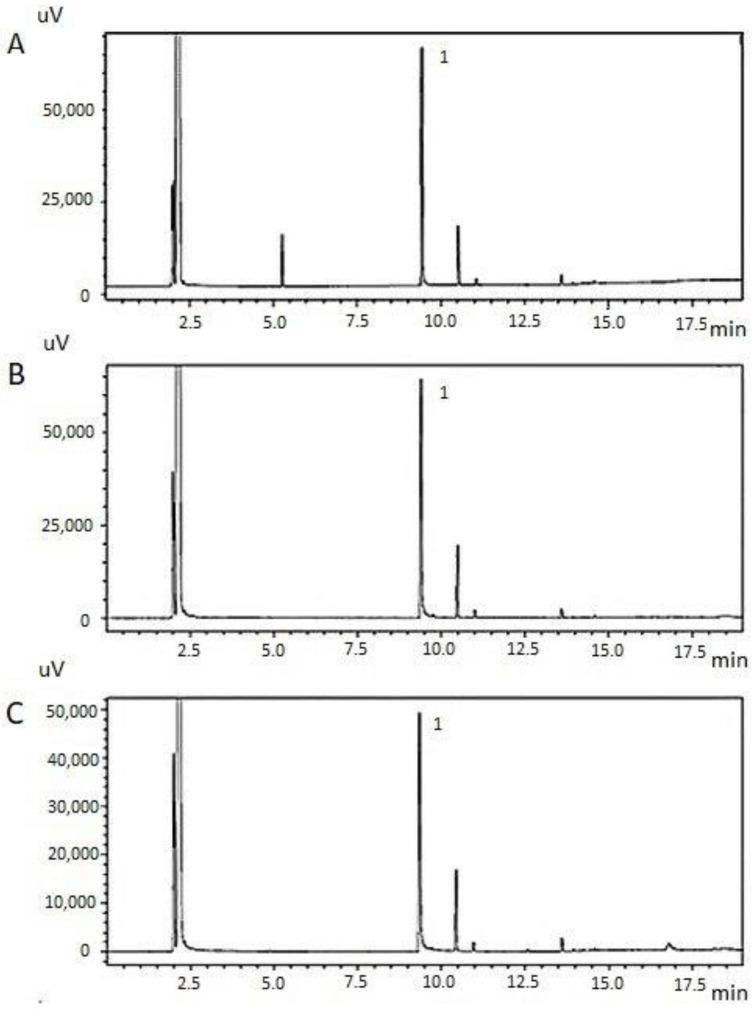
GC chromatogram of *Syzygium aromaticum* L. CO_2_ extracts obtained from different types of raw materials: (**A**)—No. 1, (**B**)—No. 2, (**C**)—No. 3; 1—eugenol.

It is known that the composition of plant extracts depends on various factors, such as the type of plant, its used part, and growing conditions [66,67]. To compare the phytochemical properties of the obtained extracts, we analysed the quantitative determination of the main biologically active substance of clove—eugenol. The study was carried out according to the European Pharmacopoeia (Ph. Eur.) using the GC/FID method [68,69]. GC chromatograms of the studied extracts are shown in Figure 3.

From the chromatogram of all samples (Figure 3), it is clear that the CO_2_ extracts contain eugenol. The analysis revealed that eugenol is a volatile product separated at a retention time of 9.40 in the chromatogram. It was eluted from a capillary GC column, having a single peak. According to the content of the main compound, clove CO_2_ extracts can be arranged in the following order: No. 2 (83.8%) > No. 1 (81.3%) > No. 3 (75.8%). The data obtained comply with the requirements of the Ph. Eur. regarding the quantitative standards of eugenol content in clove oil (75–88%) [68].

The microbiological analysis revealed the presence of an antimicrobial effect in all extracts, beginning from 0.01% concentration, against cultures of microorganisms such as *Staphylococcus aureus* ATCC 25923, *Staphylococcus epidermidis* ATCC 12228, *Enterococcus faecalis* ATCC 29212, *Escherichia coli* ATCC 25922, *Klebsiella pneumoniae* ATCC 13883, *Proteus vulgaris* ATCC 8427, *Bacillus cereus* ATCC 11778, and *Candida albicans* ATCC 10231. However, there was no statistically significant difference in the antimicrobial effect between the three samples of clove CO_2_ extracts.

Thus, taking into account the results of the extract yields and the absence of a statistically significant difference in the amount of eugenol in samples No. 1 and No. 2, from an economic point of view, we chose extract No. 1, obtained from ground whole buds of *Syzygium Aromaticum* L. before extraction, for further research.

The next step in our research was to prepare an emulsion with clove CO_2_ extract and a combination of lavender and grapefruit essential oils. It is known that the size and distribution of particles of oral formulations affect the stability, release, penetration, and bioavailability of the drug [70]. Today, a widely used high-energy method to reduce the droplet size of emulsions is ultrasonic (US) processing. In this method, the mechanical vibrations of ultrasonic waves create a sinusoidal pressure change in the emulsion system, which leads to micro-jet and shock-wave impacts and collisions between the particles and, consequently, to a reduction in their size [71,72,73].

**Figure 4:**
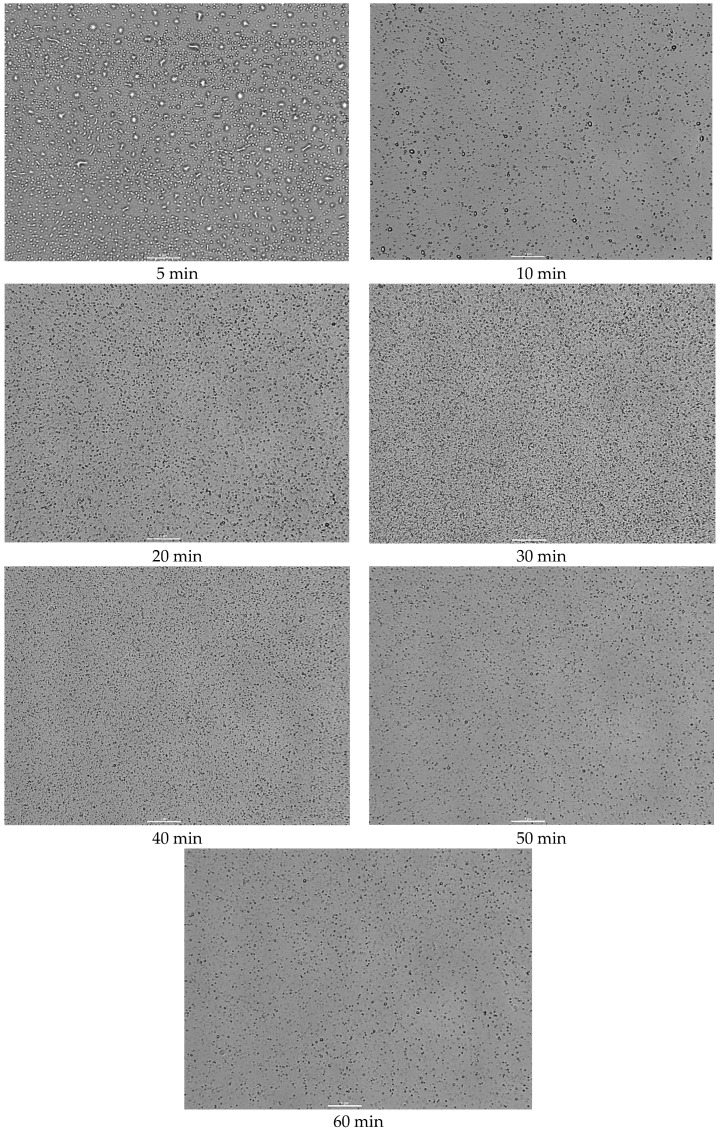
Microscopic analysis of API emulsion samples depending on the time of US exposure. Reference bar corresponds to 1 μm; 100× magnification.

**Figure 5:**
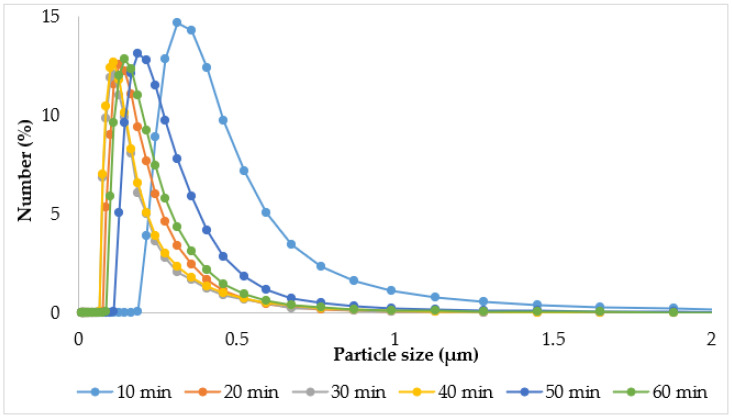
Dependence of API emulsion particle size distribution on the time of US exposure.

In addition to the particle size that is achieved during the US process, it is also important to understand the dynamic pathways to reduce processing time and avoid excessive energy delivery, which can conversely lead to particle agglomeration [74]. In order to establish a rational time for obtaining an emulsion with clove CO_2_ extract and the proposed combination of essential oils, different durations of US emulsification were used: 5, 10, 20, 30, 40, 50, and 60 min. The effect of the US exposure time on the particle size of the oil phase and the homogeneous distribution in the emulsion system was investigated by microscopic analysis (Figure 4) and laser diffraction (Figure 5 and Table 2).

As can be seen from the results (Figure 4), using a short-term sonication (5–20 min), the obtained emulsions are characterized by larger particle sizes in the oil phase. In addition, at 50 and 60 min of US exposure to the emulsions, a decrease in the number of particles and an increase in their size are observed in the field of view, in contrast to 30 and 40 min of emulsification. Thus, the long-term US exposure of emulsions with volatile components negatively affects the quality of the resulting product. This is in line with the observations of other scientists, who also show that a certain amount of energy is required to induce cavitation to minimize the droplet size in emulsions. Thus, an excess of this energy causes intense turbulence and promotes coalescence, with the formation of larger droplets [75,76,77].

The results of the microscopic analysis of the emulsion test samples correlate with the diffraction analysis data presented in Figure 5 and Table 2.

The results shown in Figure 5 show that the duration of US exposure has a significant effect on the particle size distribution of emulsions. The sample obtained after a short exposure to ultrasound (10 min) had the largest oil droplet size, which was in the range of 0.188–4.03 µm. With an increase in the time of US exposure to 20 min, the droplet size of the emulsions gradually decreased and was in the range of 0.0771–2.42 µm. It should be noted that the API emulsions’ particle size distribution values obtained at 30 and 40 min of US exposure were in the same range—from 0.0679 to 2.13 µm. Longer sonication, on the contrary, led to particle agglomeration and the formation of coarser emulsions. Thus, the particle size distribution at 50 min of US exposure ranged from 0.0876 to 3.12 µm, and at 60 min from 0.113 to 3.55 µm, respectively.

The corresponding values of the D10, D50, and D90 percentiles of all test samples are given in Table 2.

The results of Table 2 also confirm that the values of the D10, D50, and D90 percentiles gradually decreased with increasing time of US exposure from 10 to 40 min. On the contrary, exposure to ultrasound for 50 and 60 min led to an increase in these indicators. The study of Anubhav Pratap-Singh also showed that it is not necessary to constantly increase the sonication time to obtain the smallest particle size, since after a certain time, the effect of ultrasound does not have a large impact on particle size reduction [72]. Franco F.’s research also showed that the droplet size initially decreased exponentially with increasing sonication time, and then, after a certain number of minutes, tended to remain stationary [73].

Thus, based on the results of microscopic and diffraction analysis, 30 min was chosen as the rational time for obtaining API emulsions using ultrasonication, which ensures the production of particles of minimal size.

**Figure 6:**
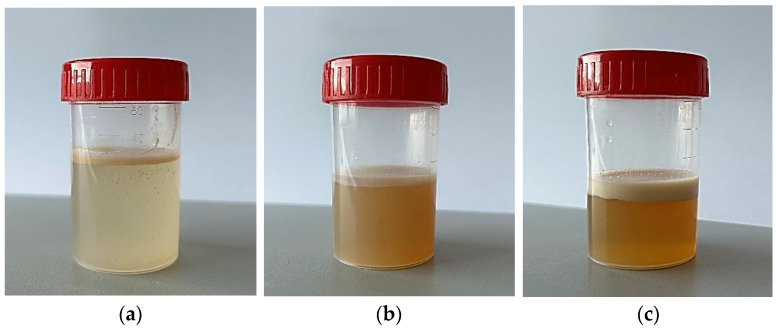
Instability of emulsions with emulsifiers: (**a**) sucrose stearate; (**b**) soybean lecithin; (**c**) sodium caseinate.

It is known that emulsions, as unstable systems, require stabilization. To ensure the kinetic and thermodynamic stability of emulsions, various surfactants are often used [78,79]. In our study, we used emulsifiers approved for oral use, namely, L-α-Lecitin soybean—3.0%, polysorbate—80–3.0%, sodium caseinate—3.0%, and sucrose stearate Sisterna^®^ SP70-C—2.0% [80,81,82]. Clove CO_2_ extract and essential oils of lavender and grapefruit were emulsified in water purified in the presence of these emulsifiers in an ultrasonic bath for 30 min. The stability of the emulsions was determined visually by the ability of the system to resist changes in physicochemical properties over time. According to the results, only the polysorbate 80 emulsifier allowed us to obtain a stable emulsion, while in other samples, when stored for 1 week, system stratification was observed (Figure 6).

An increase in the concentration of emulsifiers also did not allow for stable emulsions. At the same time, an increase in the amount of sucrose stearate led to an increase in the viscosity of the system, which did not allow the resulting system to be sprayed through a spray nozzle. An increase in the concentration of sodium caseinate and lecithin led to intense foaming both during the preparation and spraying of the emulsion. In addition, it should also be noted that despite the stability of the emulsion system, the sample with polysorbate 80 was characterized by an unpleasant odour and a very bitter taste, which are typical for this emulsifier [83,84]. These characteristics, in turn, limit the use of polysorbate 80 in the oral spray being developed.

Polymer-forming mechanical barriers are also used to stabilize emulsions [85,86,87]. Given this, to stabilize the two-phase system, it was decided to include mucoadhesive polymers as emulsifiers in the spray. By increasing the viscosity of the system, these provide improved emulsion stability and adhesion, and prolong the contact time at the application site [88,89]. Polymers can stabilize an emulsion through various mechanisms. They can act by steric hindrance, forming a shell that surrounds the oil droplets, preventing coalescence or aggregation phenomena and thus increasing the physical stability of the emulsion during storage [90]. In addition, polymers can cause an additional stabilizing effect in emulsions with hydrophobic groups, forming an adsorbed viscoelastic layer [91]. Thus, the addition of polymers to emulsion systems reduces the mobility of the continuous aqueous phase, slowing down potential destabilization processes. Therefore, the next stage of the study was devoted to the choice of mucoadhesive agent and its concentration in the spray. The main criterion for choosing the concentration of the mucoadhesive components was the ability of the polymers to form a film and a stable, homogeneous system with rational viscosity, allowing the solution to be easily sprayed through a spray nozzle.

Based on data in the literature, initially, all cellulose derivatives and sodium alginates were taken at a concentration of 2.0%, xanthan at 0.6%, and PVP and PVA at 5.0% each [92,93]. However, the concentrations taken did not allow some samples to be sprayed through the nozzle, which required reducing the concentration of polymers in the spray. The final compositions of the spray samples are shown in Table 3.

It is known that selected mucoadhesive polymers also play the role of film formers [93,94,95], providing mechanical protection for the affected areas of the mucous membrane that occur during infectious and inflammatory diseases of the oral cavity. Therefore, an indirect characteristic of oromucosal sprays with mucoadhesive agents is their ability to form film.

**Figure 7:**
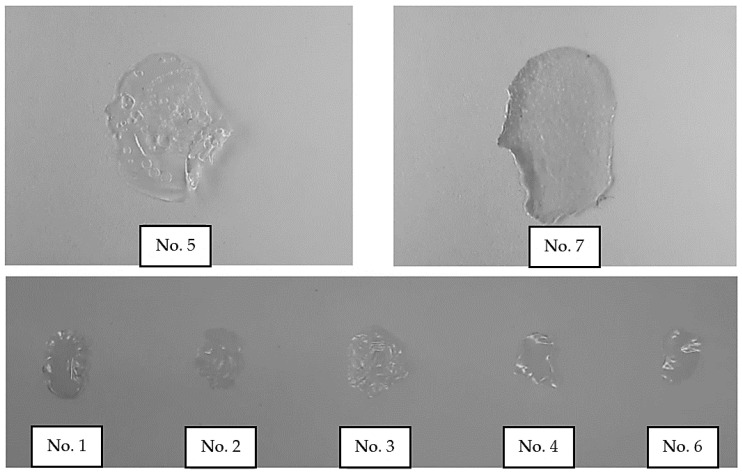
Films formed by mucoadhesive polymers: HEC (No. 1); HPC (No. 2); Na-CMC (No. 3); sodium alginate (No. 4); xanthan (No. 5); PVA (No. 6); PVP (No. 7).

The film-formation test showed that a decrease in the concentration of xanthan and PVP in sprays led to a deterioration in their film-forming ability. Films based on xanthan (No. 5) were very thin and fragile, with many small holes in the surface, while PVP (No. 7), on the contrary, formed hard glassy films with low elasticity after evaporation of the solvent (Figure 7). These characteristics limit the use of xanthan and PVP as mucoadhesive components in spray formulations. Samples No. 5 and No. 7 were excluded from our further studies.

For the remaining samples, the stability, pH, and viscosity of the sprays were examined. The results are presented in Table 4.

It is known that the pH of dental medicinal products must correspond to the pH of the oral cavity, the value of which depends on many factors and can range from 5.0 to 8.0 [41,96,97]. Based on the results (Table 4), all samples corresponded to the required pH range. The samples’ viscosity changed in the following order: No. 4 > No. 3 > No. 1 > No. 2 > No. 6. The spray based on sodium alginate (No. 4) had the highest viscosity, and the spray based on PVA (No. 6) had the lowest. However, despite its high viscosity characteristics, when checking the stability immediately after preparation and after a month of storage at a speed of 3000 rpm for 5 min, the sample based on sodium alginate (No. 4) did not pass the test, and at a speed of 10,000 rpm for 10 min, visible changes were observed in the sample based on PVA (No. 6) (Table 4). This fact can be explained by the different viscosities of the sprays and the stabilizing ability of the polymers used. The remaining spray samples were stable. Considering the results obtained, we excluded samples No. 4 and No. 6 from further studies.

The emulsion particle size distribution in the formed sprays was assessed by diffraction analysis. Changes in the particle sizes of samples No. 1–No. 3 were observed over a period of 1 month. Their physical stability was assessed by changes in the D10, D50, and D90 indicators every week. The results are shown in Table 5.

As can be seen from the results (Table 5), the sample based on HPC (No. 2) had the smallest size of oil droplets, and was characterized by the lowest viscosity of the formed system. The addition of HEC (No. 1) and Na-CMC (No. 3) led to an increase in the viscosity of the internal aqueous phase, which made it difficult to obtain smaller particle sizes during homogenization. However, as shown in Table 5, the oil droplet sizes of all samples increased with time. The HPC sample had the largest tendency to form larger droplets over time, and the HEC spray had the smallest. The obtained results can be explained by different rheological parameters of the studied samples, since with an increase in the viscosity of the dispersion medium, the particles of the dispersed phase become more stabilized, which makes it difficult for them to advance, involuntarily resulting in coalescence and aggregation. However, despite the fact that the spray with Na-CMC was characterized by the highest viscosity, the sample had the largest droplet size both after preparation and during storage for 1 month compared to the spray with HEC. According to Zhang M. et al., polymers can cause an increase in oil droplet size through a number of mechanisms: non-adsorbed emulsifiers/polymers can form multilayers around each droplet, or non-adsorbed emulsifiers/polymers can promote droplet flocculation by increasing the hydrophobicity of the droplet surface [86]. Thus, the coalescence of droplets is controlled by the ability of the stabilizer to adsorb at the interface and largely depends on the type, activity, and concentration of the polymer.

Since the textural properties of the composition also affect the consumer characteristics of the spray and, ultimately, the effectiveness of therapy, our goal was conducting a texture analysis of the studied samples, namely via cohesion, adhesion, and firmness evaluation. Back extrusion and spreadability tests were performed on a Texture Analyzer (Stable Micro Systems Ltd., Surrey, UK). The results of the back extrusion are given in Table 6.

As can be seen from the results (Table 6), the increase in compression force, which leads to deformation of the spray and, subsequently, forms the process of back extrusion, occurs in the following order: No. 2 < No. 1 < No. 3. Accordingly, this can be explained by an increase in the firmness of the spray, which is also proven by viscometrical studies (Table 4). The more liquid the consistency of the sample, the easier it is to deform, i.e., to destroy the cohesive bonds in the system. Cohesiveness ensures the uniform distribution of the spray on the mucous membrane and, at the same time, prevents its rapid washing out by saliva, allowing it to remain in the oral cavity for a longer time. The best results in terms of cohesiveness and adhesiveness were achieved by a sample of a spray based on Na-CMC (No.3). Samples based on HPC and HEC had a more liquid, flowing consistency compared to Na-CMC, and this in turn led to a decrease in their adhesive ability. By lifting the probe above the surface of the spray, the sample is separated from the disk, resulting in the measurement of the minimum drawing force of the spray. According to the data, the greater firmness (viscosity) of the Na-CMC-based spray led to an increase in the minimum force of separation of the spray from the contacting surface, which also characterizes the adhesive ability of the spray.

Javanbakht S. et al. provide data showing that, among cellulose derivatives, CMC demonstrates better bioadhesive properties and strong adhesion to biological surfaces compared to most non-ionic cellulose derivatives [53]. Shiva Golshani with co-authors also indicated that anionic polymers, which include sodium CMC, have stronger mucoadhesion force than non-ionic polymers (HEC, HPMC, HPC, MC) [46,98].

The results of the spreadability test are presented in Table 7.

According to the results in Table 7, the lowest values of the parameters under study were found in a sample of a spray based on HPC (No. 2). Samples based on HEC (No. 1) and Na-CMC (No. 3) had insignificant differences in the studied indicators of firmness, spreadability, and adhesiveness. As the firmness (viscosity) of the spray increases, the spreadability and pull-off force from the probe surface (adhesion) increase.

As is known, a feature of sprays as a dosage form is the method of extracting the drug from the package via micropumps of various design. The medicine is sprayed from the bottle through a dispenser nozzle in the form of fine particles, forming a spray torch, which allows achieving a high degree of penetration and the speedy onset of therapeutic effect. Given this, assessing the effectiveness of spraying a drug is a key aspect of spray technology.

**Figure 8:**
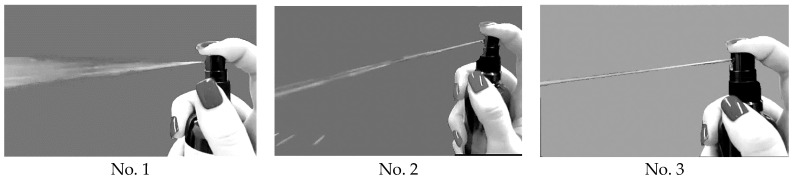
Geometry of the spray plume of samples based on HEC (No. 1), HPC (No. 2), and Na-CMC (No. 3).

**Figure 9:**
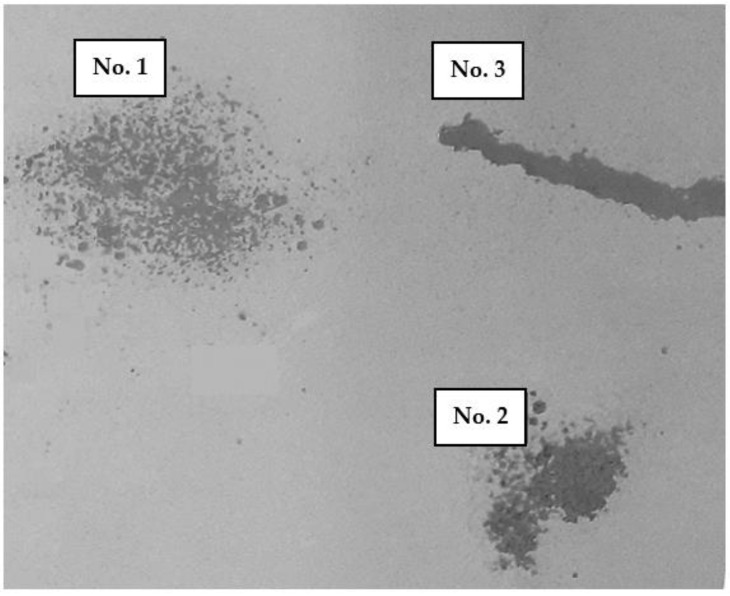
Imprint of the spray plume of samples based on HEC (No. 1), HPC (No. 2), and Na-CMC (No. 3).

In order to make the final choice of composition, we assessed the quality of spray atomization from the point of view of the geometry (structure) of the spray plume. The geometry of the spray plume was characterized by the spray angle, the structure of the spray plume, and its imprint. To undertake this, each of the test samples was sprayed once onto the substrate from the same distance (20 cm), and the results were recorded using a photo camera. The spray was released by manual pressure after a false start to the side, similar to how it is released by patients. Paper filters were used as the substrate. The results are presented in Figure 8 and Figure 9.

Visually, a significant difference in the nature of the spraying of the studied samples was noted (Figure 8 and Figure 9). Two types of spraying were clearly visible: “drip” and “jet”.

As the analysis of the data obtained has shown, the largest area of irrigated surface with a uniform distribution of finely dispersed drops of uniform size and a “full cone” shape, characteristic of the drop type of spraying, making it possible to cover most of the affected areas of the oral cavity, was created by the sample based on HEC (No. 1). The cone angle of sample No. 1 was (43 ± 3)°. The middle zone with an uneven plume imprint and a narrower asymmetric cone was created by the sample based on HPC (No. 2). The spray sample under study had a narrow spray cone angle of less than 20°, which, in turn, will provide a significantly smaller area of contact of the drug with the oral mucosa. The sample based on Na-CMC (No. 3) had a jet type of spraying in the form of one solid focused jet, resulting in a linear-point dense area with the smallest irrigated surface area.

Thus, taking into account all of the results of the experimental studies, oromucosal spray sample No. 1 was chosen as the final one. This contained 2% HEC as a mucoadhesive polymer, 0.1% sucralose and 0.2% natural grapefruit flavouring as taste additives, a combination of 0.1% potassium sorbate and 0.1% sodium benzoate as preservatives, 1.0% ethanol (96%) as a co-solvent, and purified water as a solvent.

**Figure 10:**
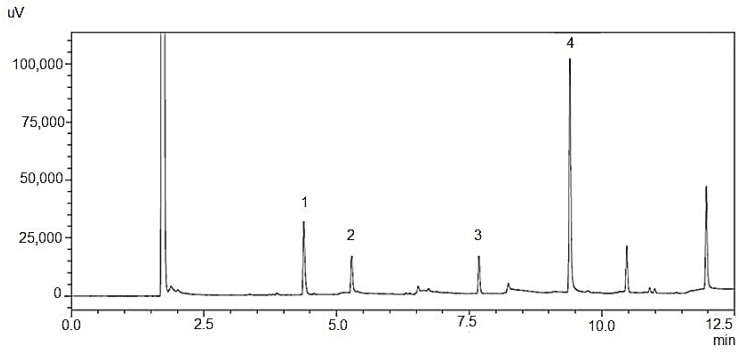
Chromatogram of spray based on HEC as mucoadhesive agent: 1—limonene; 2—linalool; 3—linalyl acetate; 4—eugenol.

The main biologically active ingredients of clove CO_2_ extract and the essential oils of lavender and grapefruit in the spray formulation with HEC as a mucoadhesive polymer were determined using the GC/FID method. Based on the chromatogram obtained, characteristic retention times were determined for the APIs of the spray as the basis for qualitative identification: limonene—4.40 min; linalool—5.31 min; linalyl acetate—7.69 min; eugenol—9.40 min. The results are presented in Figure 10.

**Figure 11:**
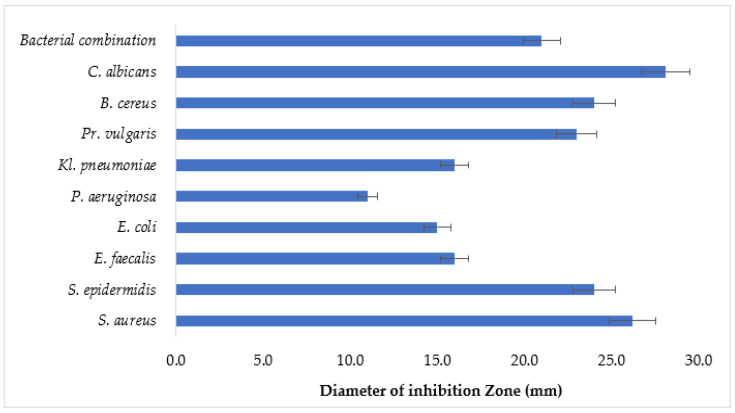
Results of the antimicrobial activity of the spray.

The antimicrobial activity of the spray based on HEC was studied in vitro by the agar disk-diffusion method, which is widely used to evaluate the antimicrobial effect of formulations containing plant-derived compounds [99]. This method is based on the ability of active substances to diffuse into agar medium which has previously been inoculated with microbial cultures. The results of the study allow us to characterize both the antimicrobial activity of the drug and the release of antimicrobial substances from the base, since the zones of inhibition of microbial growth are formed as a result of the diffusion of these substances into a dense nutrient medium. For microbiological analysis, both pure and mixed microbial cultures were used. A combination of reference microorganisms was prepared for polymicrobial analysis: *S. aureus* + *S. epidermidis* + *E. faecalis* + *S. mutans* + *S. mitis* + *E. coli*. The results were recorded by measuring the zone of inhibition of microbial growth (mm). The results are shown in Figure 11.

The experimental results presented in Figure 11 indicate that the developed spray, comprising a combination of clove CO_2_ extract and essential oils of lavender and grapefruit, has a wide spectrum of antimicrobial action against all used cultures of bacteria and fungus of the genus *Candida*. Considering the scale of the inhibition zone, the oromucosal spray had the strongest inhibitory effect on *C. albicans* (28.1 ± 0.3 mm) and *S. aureus* (26.2 ± 0.2 mm), with the order of sensitivity of the other strains as follows: *S. epidermidis* > *B. cereus* > *Pr. vulgaris* > *Kl. pneumoniae* > *E. faecalis* > *E. coli* > *P. aeruginosa*. The spray also showed similar sensitive behaviour to a combination of microbial cultures, demonstrating pronounced bactericidal sensitivity (21.3 ± 0.5 mm). In addition, it can be argued that the viscosity of the spray formed by the HEC does not interfere with the release of APIs from the finished product, which, accordingly, will ensure its high bioavailability.

Considering that two-phase systems are prone to destabilization, the purpose of our further research was to determine the oromucosal spray’s storage stability. The developed product with plant-derived compounds was stored in two different conditions: in a climatic chamber (40 ± 2 °C/75 ± 5% RH) and at room temperature (25 ± 2 °C/60 ± 5% RH) for 3 months of storage [100]. Quality parameters (sensory properties, uniformity, pH value, centrifugation stability test, viscous characteristics, API content) were evaluated.

The organoleptic properties of the spray and the pH value during storage remained unchanged in the two temperature regimes. It passed the colloidal stability test and no phase separation or other changes were observed. During the control period of storage, the quantitative content of APIs was also determined, remaining within the permissible limit of 5 percent. The viscosity characteristics of the spray also did not undergo significant changes.

Therefore, based on the conducted research, it was established that throughout the entire storage period at two temperature regimes, the developed oromucosal spray with CO_2_ extract and essential oils of lavender and grapefruit remained physically, chemically, and technologically stable for 3 months.

## 4. Conclusions

In this research, oromucosal spray containing a combination of clove CO_2_ extract and essential oils of lavender and grapefruit as APIs was developed and evaluated for the first time. These plant-derived compounds exhibit anti-inflammatory, antibacterial, reparative, and analgesic properties that are suitable for the effective treatment of infectious and inflammatory diseases of the periodontium and the mucous membrane of the oral cavity.

According to the yield value and the results of the chemical and microbiological analyses, the type of raw material for obtaining clove CO_2_ extract was chosen. Based on the study of the size and homogeneity of the API particle distribution, a rational duration for emulsion production using ultrasonic exposure was established.

Emulsion stability depended on the type of mucoadhesive polymer and their amount. The sprays based on cellulose derivatives—HEC, HPC, and Na-CMC—had the best quality parameters. With increasing firmness (viscosity) of the system, the spreadability, cohesive, and adhesive properties of the spray were improved, but at the same time spraying quality was decreased, which, in turn, resulted in a small contact area of the drug with the oral mucosa. Considering viscous and textural characteristics, as well as film-forming, adhesive, and spraying ability, HEC was chosen as the optimal mucoadhesive polymer for the oromucosal spray formulation. It will allow the drug to remain in the oral cavity for a more prolonged period and form a protective film on the affected areas of soft tissue and mucous, isolating external irritants.

The identification of clove CO_2_ extract and essential oils of lavender and grapefruit in the spray was carried out by the GC/FID method.

High antimicrobial activity of the developed spray against a wide range of microorganisms was established, which indicates its potential as a medicine to prevent infections and, accordingly, treat infectious and inflammatory diseases of the oral cavity.

Future research will focus on elucidating spray bioavailability to characterize the release of active ingredients, and on conducting pharmacological tests. Additionally, ongoing stability studies will be extended to assess the developed composition over a longer period.

## Figures and Tables

**Table 1:** Information about ingredients used in the oromucosal spray formulation.

Ingredient	Manufacturer	Function
Clove CO_2_ extract	UAB “Eno extractum”, Kaunas, Lithuania	API
Lavender essential oil	Sigma-Aldrich, Sofia, Bulgaria	API
Grapefruit essential oil	SAFC Supply Solutions, St Saint Louis, MI, USA	API
Ethanol 96%	AB “Vilniaus degtinė”, Vilnius, Lithuania	Co-solvent, preservative
Sucralose	ThermoFisher, Kandel, Germany	Sweetener
Sodium benzoate	Sigma-Aldrich, Amsterdam, The Netherlands	Preservative
Potassium sorbate	Sigma-Aldrich, St Saint Louis, MI, USA	Preservative
Grapefruit powder flavour	Xi’an Taima Biological Engineering Co. Ltd, Xian, China	Flavouring
HEC HPC Sodium CMC Sodium alginate Xanthan PVP PVA	Sigma-Aldrich, St Saint Louis, Missouri, USA Alfa Aesar GmbH & Co, Karlsruhe, Germany Sigma-Aldrich, Helsinki, Finland Carl Roth GmbH + Co, Karlsruhe, Germany Sigma-Aldrich, St Saint Louis, MI, USA Sigma-Aldrich, St Saint Louis, MI, USA Sigma-Aldrich, St Saint Louis, MI, USA	Mucoadhesive agent, viscosifier, film former, stabilizer
Purified water	Ph. Eur. 01/2008:0008, LSMU laboratory, Kaunas, Lithuania	Solvent

**Table 2:** Percentile (D10, D50, D90) values of API emulsion production with different times of US exposure (n = 3, ±SD).

Duration of US Emulsification	D10 (µm)	D50 (µm)	D90 (µm)
10 min	0.265 ± 0.015	0.389 ± 0.019	0.704 ± 0.026
20 min	0.106 ± 0.013	0.165 ± 0.015	0.321 ± 0.023
30 min	0.091 ± 0.010	0.143 ± 0.012	0.302 ± 0.015
40 min	0.090 ± 0.009	0.139 ± 0.010	0.298 ± 0.020
50 min	0.119 ± 0.020	0.183 ± 0.017	0.353 ± 0.025
60 min	0.156 ± 0.022	0.237 ± 0.021	0.436 ± 0.028

**Table 3:** Compositions of experimental spray samples.

Ingredient	Sample No./Amount, %						
	1	2	3	4	5	6	7
Clove CO_2_ extract	0.5	0.5	0.5	0.5	0.5	0.5	0.5
Lavender essential oil	0.2	0.2	0.2	0.2	0.2	0.2	0.2
Grapefruit essential oil	0.3	0.3	0.3	0.3	0.3	0.3	0.3
Ethanol 96%	1.0	1.0	1.0	1.0	1.0	1.0	1.0
Sucralose	0.1	0.1	0.1	0.1	0.1	0.1	0.1
Sodium benzoate	0.1	0.1	0.1	0.1	0.1	0.1	0.1
Potassium sorbate	0.1	0.1	0.1	0.1	0.1	0.1	0.1
Grapefruit powder flavour	0.2	0.2	0.2	0.2	0.2	0.2	0.2
HEC	2.0						
HPC		1.5					
Na-CMC			1.5				
Sodium alginate				1.5			
Xanthan					0.4		
PVA						3.0	
PVP							3.0
Purified water	Up to 100.00	Up to 100.00	Up to 100.00	Up to 100.00	Up to 100.00	Up to 100.00	Up to 100.00

**Table 4:** Structural viscosity, pH, and colloidal stability of sprays (n = 3, ±SD).

Sample No. (Type of Mucoadhesive Polymer)	pH	Structural Viscosity, mPa·s	Stability (After Centrifugation)
No. 1 (HEC)	5.824 ± 0.021	124.4 ± 5.5	+
No. 2 (HPC)	5.985 ± 0.003	105.8 ± 2.9	+
No. 3 (Na-CMC)	6.632 ± 0.006	190.3 ± 9.0	+
No. 4 (Sodium alginate)	6.373 ± 0.045	342.2 ± 9.2	–
No. 6 (PVA)	6.093 ± 0.015	29.1 ± 3.6	–

**Table 5:** Percentile (D10, D50, D90) values of spray samples within 1 month of storage (n = 3, ±SD).

Storage Period	Sample No. (Type of Mucoadhesive Polymer)								
	No. 1 (HEC)			No. 2 (HPC)			No. 3 (Na-CMC)		
	D10 (µm)	D50 (µm)	D90 (µm)	D10 (µm)	D50 (µm)	D90 (µm)	D10 (µm)	D50 (µm)	D90 (µm)
0 week	0.372 ± 0.005	0.501 ± 0.006	1.300 ± 0.001	0.287 ± 0.002	0.392 ± 0.004	0.670 ± 0.005	0.388 ± 0.004	0.559 ± 0.006	1.500 ± 0.002
1 week	0.393 ± 0.004	0.564 ± 0.005	1.380 ± 0.003	0.303 ± 0.003	0.434 ± 0.005	0.702 ± 0.004	0.416 ± 0.002	0.587 ± 0.005	1.610 ± 0.002
2 weeks	0.411 ± 0.006	0.592 ± 0.004	1.490 ± 0.002	0.334 ± 0.004	0.486 ± 0.005	0.743 ± 0.006	0.448 ± 0.005	0.628 ± 0.006	1.680 ± 0.004
3 weeks	0.456 ± 0.005	0.649 ± 0.005	1.830 ± 0.003	0.465 ± 0.006	0.594 ± 0.003	1.040 ± 0.003	0.454 ± 0.003	0.658 ± 0.005	1.860 ± 0.005
4 weeks	0.504 ± 0.006	0.706 ± 0.006	1.910 ± 0.002	0.484 ± 0.005	0.630 ± 0.006	1.160 ± 0.004	0.517 ± 0.005	0.772 ± 0.006	2.320 ± 0.006

**Table 6:** Back extrusion of sprays (n = 3, ±SD).

Sample No. (Type of Mucoadhesive Polymer)	Maximum Compressing Force, g	Cohesiveness, g·sec	Maximum Retracting Force, g	Adhesiveness, g·sec
No. 1 (HEC)	13.77 ± 0.40	54.76 ± 0.58	11.31 ± 0.14	1.97 ± 0.13
No. 2 (HPC)	11.09 ± 0.38	53.03 ± 0.18	7.83 ± 0.26	0.89 ± 0.06
No. 3 (Na-CMC)	14.07 ± 0.31	58.20 ± 0.53	12.25 ± 0.14	3.44 ± 0.14

**Table 7:** Spreadability of sprays (n = 3, ±SD).

Sample No. (Type of Mucoadhesive Polymer)	Firmness, g	Spreadability, g·sec	Adhesive Force, g	Adhesiveness, g·sec
No. 1 (HEC)	7.957 ± 0.639	3.797 ± 0.223	5.216 ± 1.015	1.237 ± 0.768
No. 2 (HPC)	7.133 ± 1.422	3.138 ± 1.280	3.945 ± 0.981	1.231 ± 0.287
No. 3 (Na-CMC)	8.410 ± 1.066	3.816 ± 0.242	6.510 ± 1.150	2.224 ± 0.235

## Data Availability

The original contributions presented in the study are included in the article, further inquiries can be directed to the corresponding author.

## References

[1] Peres M.A., Macpherson L.M.D., Weyant R.J., Daly B., Venturelli R., Mathur M.R., Listl S., Celeste R.K., Guarnizo-Herreño C.C., Kearns C. (2019). Oral diseases: A global public health challenge. Lancet.

[2] Laudenbach J.M., Kumar S.S. (2020). Common Dental and Periodontal Diseases. Dermatol. Clin..

[3] Hajishengallis G., Chavakis T. (2021). Local and systemic mechanisms linking periodontal disease and inflammatory comorbidities. Nat. Rev. Immunol..

[4] Bui F.Q., Almeida-da-Silva C.L.C., Huynh B., Trinh A., Liu J., Woodward J., Asadi H., Ojcius D.M. (2019). Association between periodontal pathogens and systemic disease. Biomed. J..

[5] Kinane D.F., Stathopoulou P.G., Papapanou P.N. (2017). Periodontal diseases. Nat. Rev. Dis. Primers.

[6] Visentin D., Gobin I., Maglica Ž. (2023). Periodontal Pathogens and Their Links to Neuroinflammation and Neurodegeneration. Microorganisms.

[7] Chinsembu K.C. (2016). Plants and other natural products used in the management of oral infections and improvement of oral health. Acta Trop..

[8] Moghadam E.T., Yazdanian M., Tahmasebi E., Tebyanian H., Ranjbar R., Yazdanian A., Seifalian A., Tafazoli A. (2020). Current herbal medicine as an alternative treatment in dentistry: In vitro, in vivo and clinical studies. Eur. J. Pharmacol..

[9] Arumugam B., Subramaniam A., Alagaraj P. (2020). A Review on Impact of Medicinal Plants on the Treatment of Oral and Dental Diseases. Cardiovasc. Hematol. Agents Med. Chem..

[10] Pulikkotil S.J., Nath S. (2015). Potential of clove of *Syzygium aromaticum* in development of a therapeutic agent for periodontal disease. A review. S. Afr. Dent. J..

[11] Batiha G.E., Alkazmi L.M., Wasef L.G., Beshbishy A.M., Nadwa E.H., Rashwan E.K. (2020). *Syzygium aromaticum* L. *(Myrtaceae):* Traditional Uses, Bioactive Chemical Constituents, Pharmacological and Toxicological Activities. Biomolecules.

[12] Askari V.R., Najafi Z., Rahimi V.B. (2023). Syzygium aromaticum—Role in Oral Health and Dental Care September 2023. Pharmacological Studies in Natural Oral Care.

[13] Haro-González J.N., Castillo-Herrera G.A., Martínez-Velázquez M., Espinosa-Andrews H. (2021). Clove Essential Oil (*Syzygium aromaticum* L. *Myrtaceae*): Extraction, Chemical Composition, Food Applications, and Essential Bioactivity for Human Health. Molecules.

[14] Mendi A., Yağci B.G., Kiziloğlu M., Saraç N., Yilmaz D., Uğur A., Uçkan D. (2017). Effects of *Syzygium aromaticum, Cinnamomum zeylanicum*, and *Salvia triloba* extracts on proliferation and differentiation of dental pulp stem cells. J. Appl. Oral Sci..

[15] Shahbazi Y. (2019). Antioxidant, antibacterial, and antifungal properties of nanoemulsion of clove essential oil. Nanomedicine Res. J..

[16] Danthu P., Simanjuntak R., Fawbush F., Leong P.T.J.M., Razafimamonjison G., Abdillahi M.M., Jahiel M., Penot E. (2020). The clove tree and its products (clove bud, clove oil, eugenol): Prosperous today but what of tomorrow’s restrictions?. Fruits.

[17] Kaur K., Kaushal S., Rani R. (2019). Chemical composition, antioxidant and antifungal potential of clove (*Syzygium aromaticum*) essential oil, its major compound and its derivatives. J. Essent. Oil Bear. Plants.

[18] Sueksakit K., Thisayakorn K., Khueynok V., Sriyam K., Pahusee D., Buddhakala N. (2013). Preliminary study of *Syzygium aromaticum* L. on analgesic activity in rats. Thai J. Pharm. Sci..

[19] AL-Mahdi Z.K.A., Witwit L.J., Ubaid I.A. (2021). Activity of Cloves, Cinnamon and Thyme Essential Oils Against Some Oral Bacteria. Al-Kitab. J. for Pure Sci..

[20] Pramod K., Ansari S.H., Ali J. (2010). Eugenol: A natural compound with versatile pharmacological actions. Nat. Prod. Commun..

[21] Frohlich P.C., Santos K.A., Palú F., Cardozo-Filho L., da Silva C., da Silva E.A. (2019). Evaluation of the effects of temperature and pressure on the extraction of eugenol from clove (*Syzygium aromaticum* L.) leaves using supercritical CO_2_. J. Supercrit. Fluids.

[22] Pandey V.K., Srivastava S., Ashish, Dash K.K., Singh R., Dar A.H., Singh T., Farooqui A., Shaikh A.M., Kovacs B. (2023). Bioactive properties of clove (Syzygium aromaticum) essential oil nanoemulsion: A comprehensive review. Heliyon.

[23] Aboelmaati M.F., Mahgoub S., Labib S., Al-Gaby A.M.A., Fawzy Ramadan M. (2016). Phenolic extracts of clove (*Syzygium aromaticum*) with novel antioxidant and antibacterial activities. Eur. J. Integr. Med..

[24] Martins R., Barbosa A., Advinha B., Sales H., Pontes R., Nunes J. (2023). Green Extraction Techniques of Bioactive Compounds: A State-of-the-Art Review. Processes.

[25] Peana A.T., D’Aquila P.S., Panin F., Serra G., Pippia P., Moretti M.D. (2002). Anti-inflammatory activity of linalool and linalyl acetate constituents of essential oils. Phytomedicine.

[26] Kajjari S., Joshi R.S., Hugar S.M., Gokhale N., Meharwade P., Uppin C. (2022). The Effects of Lavender Essential Oil and its Clinical Implications in Dentistry: A Review. Int. J. Clin. Pediatr. Dent..

[27] Ait Said L., Zahlane K., Ghalbane I., El Messoussi S., Romane A., Cavaleiro C., Salgueiro L. (2015). Chemical composition and antibacterial activity of *Lavandula coronopifolia* essential oil against antibiotic-resistant bacteria. Nat. Prod. Res..

[28] Zuzarte M., Gonçalves M.J., Cruz M.T., Cavaleiro C., Canhoto J., Vaz S., Pinto E., Salgueiro L. (2012). *Lavandula luisieri* essential oil as a source of antifungal drugs. Food Chem..

[29] Pathan J.M., Dadpe M.V., Kale Y.J., Dahake P.T., Kendre S.B. (2020). Evaluation of lavender oil as a topical analgesic agent before dental anaesthesia through pain rating scales—An in vivo study. IOSR J. Dent. Med. Sci..

[30] Medeleanu M.L., Fărcaș A.C., Coman C., Leopold L., Diaconeasa Z., Socaci S.A. (2023). Citrus essential oils—Based nano-emulsions: Functional properties and potential applications. Food Chem. X.

[31] Radu C.M., Radu C.C., Bochiș S.A., Arbănași E.M., Lucan A.I., Murvai V.R., Zaha D.C. (2023). Revisiting the Therapeutic Effects of Essential Oils on the Oral Microbiome. Pharmacy.

[32] Potocka W., Assy Z., Bikker F.J., Laine M.L. (2023). Current and Potential Applications of Monoterpenes and Their Derivatives in Oral Health Care. Molecules.

[33] Denkova-Kostova R., Teneva D., Tomova T., Goranov B., Denkova Z., Shopska V., Slavchev A., Hristova-Ivanova Y. (2020). Chemical composition, antioxidant and antimicrobial activity of essential oils from tangerine (*Citrus reticulata* L.), grapefruit (*Citrus paradisi* L.), lemon (*Citrus lemon* L.) and cinnamon (*Cinnamomum zeylanicum Blume*). Z. Naturforsch. C J. Biosci..

[34] Okunowo W., Oyedeji O., Afolabi L., Matanmi E. (2013). Essential Oil of Grape Fruit (*Citrus paradisi*) Peels and Its Antimicrobial Activities. Am. J. Plant Sci..

[35] Churata-Oroya D.E., Ramos-Perfecto D., Moromi-Nakata H., Martínez-Cadillo E., Castro-Luna A., Garcia-de-la-guarda R. (2016). Efecto antifúngico de Citrus paradisi “toronja” sobre cepas de Candida albicans aisladas de pacientes con estomatitis subprotésica. Rev. Estomatol. Herediana.

[36] Gugulethu M., Mongikazi N., Opeoluwa O., Mavuto G., Adebola O. (2021). Chemical Profiling, Toxicity and Anti-Inflammatory Activities of Essential Oils from Three Grapefruit Cultivars from KwaZulu-Natal in South Africa. Molecules.

[37] Deng W., Liu K., Cao S., Sun J., Zhong B., Chun J. (2020). Chemical Composition, Antimicrobial, Antioxidant, and Antiproliferative Properties of Grapefruit Essential Oil Prepared by Molecular Distillation. Molecules.

[38] Nakajima M., Tanner E.E.L., Nakajima N., Ibsen K.N., Mitragotri S. (2021). Topical treatment of periodontitis using an iongel. Biomaterials.

[39] Nittayananta W., Limsuwan S., Srichana T., Sae-Wong C., Amnuaikit T. (2018). Oral spray containing plant-derived compounds is effective against common oral pathogens. Arch. Oral Biol..

[40] Srisatjaluk R.L., Klongnoi B., Wongsirichat N. (2016). Antimicrobial effect of topical local anesthetic spray on oral microflora. J. Dent. Anesth. Pain Med..

[41] Sanguansajapong V., Sakdiset P., Puttarak P. (2022). Development of Oral Microemulsion Spray Containing Pentacyclic Triterpenes-Rich *Centella asiatica* (L.) Urb. Extract for Healing Mouth Ulcers. Pharmaceutics.

[42] Sharma R., Kumar S., Malviya R., Prajapati Bhupendra G., Puri D., Limmatvapirat S., Sriamornsak P. (2024). Recent advances in biopolymer-based mucoadhesive drug delivery systems for oral application. J. Drug Deliv. Technol..

[43] Paderini C., Compilato D., Giannola L.I., Campisi G. (2012). Oral local drug delivery and new perspectives in oral drug formulation. Oral Surg. Oral Med. Oral Pathol. Oral Radiol..

[44] Bruschi M.L., De Freitas O. (2005). Oral bioadhesive drug delivery systems. Drug Dev. Ind. Pharm..

[45] Russo E., Selmin F., Baldassari S., Gennari C.G.M., Caviglioli G., Cilurzo F., Minghetti P., Parodi B. (2017). A focus on mucoadhesive polymers and their application in buccal dosage forms. Int. J. Biol. Macromol..

[46] Golshani S., Vatanara A., Amin M. (2022). Recent Advances in Oral Mucoadhesive Drug Delivery. J. Pharm. Pharm. Sci..

[47] Mansuri S., Kesharwani P., Jain K., Tekade R.K., Jain N.K. (2016). Mucoadhesion: A promising approach in drug delivery system. React. Funct. Polym..

[48] Kapoor D., Patel M., Vyas R.B., Lad C., Lal B. (2015). Site Specific drug delivery through nasal route using bioadhesive polymers. J. Drug Deliv. Ther..

[49] Dubashynskaya N.V., Petrova V.A., Skorik Y.A. (2024). Biopolymer Drug Delivery Systems for Oromucosal Application: Recent Trends in Pharmaceutical R&D. Int. J. Mol. Sci..

[50] Akca G., Ozdemir A., Oner Z.G., Senel S. (2018). Comparison of different types and sources of chitosan for the treatment of infections in the oral cavity. Res. Chem. Intermediat..

[51] Casale M., Moffa A., Vella P., Sabatino L., Capuano F., Salvinelli B., Lopez A.M., Carinci F., Salvinelli F. (2016). Hyaluronic acid: Perspectives in dentistry. A systematic review. Int. J. Immunopathol. Pharmacol..

[52] Jadav M., Pooja D., Adams D.J., Kulhari H. (2023). Advances in Xanthan Gum-Based Systems for the Delivery of Therapeutic Agents. Pharmaceutics.

[53] Javanbakht S., Shaabani A. (2019). Carboxymethyl cellulose-based oral delivery systems. Int. J. Biol. Macromol..

[54] Majumder T., Biswas G.R., Majee S.B. (2016). Hydroxy Propyl Methyl Cellulose: Different Aspects in Drug Delivery. J. Pharm. Pharmacol..

[55] Brannigan R.P., Khutoryanskiy V.V. (2019). Progress and current trends in the synthesis of novel polymers with enhanced mucoadhesive properties. Macromol. Biosci..

[56] Alawdi S., Solanki A.B. (2021). Mucoadhesive Drug Delivery Systems: A Review of Recent Developments. J. Sci. Res. Med. Biol. Sci..

[57] Puri V., Sharma A., Maman P., Rathore N., Singh I. (2019). Overview of mucoadhesive biopolymers for buccal drug delivery systems. Int. J. App. Pharm..

[58] Jabeen N., Atif M. (2024). Polysaccharides based biopolymers for biomedical applications: A review. Polym. Adv. Technol..

[59] Raghav N., Vashisth C., Mor N., Arya P., Sharma M.R., Kaur R., Bhatti S.P., Kennedy J.F. (2023). Recent advances in cellulose, pectin, carrageenan and alginate-based oral drug delivery systems. Int. J. Biol. Macromol..

[60] Madrazo-Jiménez M., Rodríguez-Caballero Á., Serrera-Figallo M., Garrido-Serrano R., Gutiérrez-Corrales A., Gutiérrez-Pérez J.L., Torres-Lagares D. (2016). The effects of a topical gel containing chitosan, 0.2% chlorhexidine, allantoin and despanthenol on the wound healing process subsequent to impacted lower third molar extraction. Med. Oral Patol. Oral Cir. Bucal..

[61] Szekalska M., Wróblewska M., Trofimiuk M., Basa A., Winnicka K. (2019). Alginate oligosaccharides affect mechanical properties and antifungal activity of alginate buccal films with posaconazole. Mar. Drugs.

[62] Pamlényi K., Kristó K., Jójárt-Laczkovich O., Regdon G. (2021). Formulation and optimization of sodium alginate polymer film as a buccal mucoadhesive drug delivery system containing cetirizine dihydrochloride. Pharmaceutics.

[63] Elbanna S.A., Ebada H.M., Abdallah O.Y., Essawy M.M., Abdelhamid H.M., Barakat H.S. (2023). Novel tetrahydrocurcumin integrated mucoadhesive nanocomposite κ-carrageenan/xanthan gum sponges: A strategy for effective local treatment of oral cancerous and precancerous lesions. Drug Deliv..

[64] Tonglairoum P., Ngawhirunpat T., Rojanarata T., Panomsuk S., Kaomongkolgit R., Opanasopit P. (2015). Fabrication of mucoadhesive chitosan coated polyvinylpyrrolidone/cyclodextrin/clotrimazole sandwich patches for oral candidiasis. Carbohydr. Polym..

[65] Šernaitė L., Rasiukevičiūtė N., Dambrauskienė E., Viškelis P., Valiuškaitė A. (2020). Efficacy of plant extracts and essential oils for biocontrol of strawberry pathogen Botrytis cinerea. Zemdirb. Agric..

[66] Köse Y.B., Karahisar E., İşcan G., Kürkçüoğlu M., Tugay O. (2021). Chemical Composition and Anticandidal Activity of Essential Oils Obtained From Different Part of *Prangos heyniae H*. Duman & M. F. Watson. Rec. Nat. Prod..

[67] Alfikri F.N., Pujiarti R., Wibisono M.G., Hardiyanto E.B. (2020). Yield, Quality, and Antioxidant Activity of Clove (*Syzygium aromaticum* L.) Bud Oil at the Different Phenological Stages in Young and Mature Trees. Scientifica.

[68] (2016). European Pharmacopeia 9.0.

[69] Sruthi B.Y.K., Gurupadayya B.M., Venkata Sairam K., Narendra Kumar T. (2014). Development and validation of GC method for the estimation of eugenol in clove extract. Int. J. Pharm. Pharm. Sci..

[70] Mok Z.H. (2023). The effect of particle size on drug bioavailability in various parts of the body. Adv. Pharm. J..

[71] Gupta A., Eral B.H., Hatton A.T., Doyle P.S. (2015). Controlling and predicting droplet size of nanoemulsions: Scaling relations with experimental validation. Soft Matter..

[72] Pratap-Singh A., Guo Y., Lara Ochoa S., Fathordoobady F., Singh A. (2021). Optimal ultrasonication process time remains constant for a specific nanoemulsion size reduction system. Sci. Rep..

[73] Franco F., Pérez-Maqueda L.A., Pérez-Rodríguez J.L. (2004). The effect of ultrasound on the particle size and structural disorder of a well-ordered kaolinite. J. Colloid. Interface Sci..

[74] Siva S.P., Ho Y., Kow K.-W., Chan C.-H., Tang S. (2018). Prediction of droplet sizes for oil-in-water emulsion systems assisted by ultrasound cavitation: Transient scaling law based on dynamic breakup potential. Ultrason. Sonochem..

[75] Ali H.S.M., Ahmed S.A., Alqurshi A.A., Alalawi A.M., Shehata A.M., Alahmadi Y.M. (2022). Boosting Tadalafil Bioavailability via Sono-Assisted Nano-Emulsion-Based Oral Jellies: Box–Behnken Optimization and Assessment. Pharmaceutics.

[76] Sinsuebpol C., Changsan N. (2020). Effects of ultrasonic operating parameters and emulsifier system on sacha inchi oil nanoemulsion characteristics. J. Oleo Sci..

[77] Leong T.S., Wooster T.J., Kentish S.E., Ashokkumar M. (2009). Minimising oil droplet size using ultrasonic emulsification. Ultrason. Sonochem..

[78] McClements D.J., Jafari S.M. (2018). Improving emulsion formation, stability and performance using mixed emulsifiers: A review. Adv. Colloid Interface Sci..

[79] Marium F., Muhammad A.S., Sofia A., Sadia H.K., Iqbal A. (2014). Emulsion Separation, Classification and Stability Assessment. RADS J. Pharm. Allied Health Sci..

[80] Matulyte I., Kasparaviciene G., Bernatoniene J. (2020). Development of New Formula Microcapsules from Nutmeg Essential Oil Using Sucrose Esters and Magnesium Aluminometasilicate. Pharmaceutics.

[81] Martin M.J., Trujillo L.A., Garcia M.C., Alfaro M.C., Muñoz J. (2018). Effect of emulsifier HLB and stabilizer addition on the physical stability of thyme essential oil emulsions. J. Dispers. Sci. Technol..

[82] Riquelme N., Sepúlveda C., Arancibia C. (2020). Influence of Ternary Emulsifier Mixtures on Oxidative Stability of Nanoemulsions Based on Avocado Oil. Foods.

[83] Castañeda Ruiz A.J., Shetab Boushehri M.A., Phan T., Carle S., Garidel P., Buske J., Lamprecht A. (2022). Alternative Excipients for Protein Stabilization in Protein Therapeutics: Overcoming the Limitations of Polysorbates. Pharmaceutics.

[84] Coupland J.N., Hayes J.E. (2014). Physical approaches to masking bitter taste: Lessons from food and pharmaceuticals. Pharm. Res..

[85] de Carvalho-Guimarães F.B., Correa K.L., de Souza T.P., Rodríguez A.J.R., Ribeiro-Costa R.M., Silva-Júnior J.O.C. (2022). A Review of Pickering Emulsions: Perspectives and Applications. Pharmaceuticals.

[86] Zhang M., Yang B., Liu W., Li S. (2017). Influence of hydroxypropyl methylcellulose, methylcellulose, gelatin, poloxamer 407 and poloxamer 188 on the formation and stability of soybean oil-in-water emulsions. Asian J. Pharm. Sci..

[87] Smoleński M., Karolewicz B., Gołkowska A.M., Nartowski K.P., Małolepsza-Jarmołowska K. (2021). Emulsion-Based Multicompartment Vaginal Drug Carriers: From Nanoemulsions to Nanoemulgels. Int. J. Mol. Sci..

[88] Kumar R., Islam T., Nurunnabi M. (2022). Mucoadhesive carriers for oral drug delivery. J. Control Release.

[89] Shahadha R.W., Maraie N.K. (2023). Mucoadhesive Film Forming Spray for Buccal Drug Delivery: A Review. Al Mustansiriyah J. Pharm. Sci..

[90] Whistler R.L. (2012). Industrial Gums: Polysaccharides and Their Derivatives.

[91] Dickinson E. (1997). Properties of emulsions stabilized with milk proteins: Overview of some recent developments. J. Dairy Sci..

[92] Bakhrushina E., Anurova M., Demina N., Kashperko A., Rastopchina O., Bardakov A., Krasnyuk I. (2020). Comparative Study of the Mucoadhesive Properties of Polymers for Pharmaceutical Use. Open Access Maced. J. Med. Sci..

[93] Umar A.K., Butarbutar M., Sriwidodo S., Wathoni N. (2020). Film-Forming Sprays for Topical Drug Delivery. Drug Des. Dev. Ther..

[94] Boddupalli B.M., Mohammed Z.N., Nath R.A., Banji D. (2010). Mucoadhesive drug delivery system: An overview. J. Adv. Pharm. Technol. Res..

[95] Mukhopadhyay R., Gain S., Verma S., Singh B., Vyas M., Mehta M., Haque A. (2018). Polymers in designing the mucoadhesive films: A comprehensive review. Int. J. Green Pharm..

[96] Baliga S., Muglikar S., Kale R. (2013). Salivary pH: A diagnostic biomarker. J. Indian Soc. Periodontol..

[97] Maslii Y., Ruban O., Kasparaviciene G., Kalveniene Z., Materiienko A., Ivanauskas L., Mazurkeviciute A., Kopustinskiene D.M., Bernatoniene J. (2020). The Influence of pH Values on the Rheological, Textural and Release Properties of Carbomer Polacril^®^ 40P-Based Dental Gel Formulation with Plant-Derived and Synthetic Active Components. Molecules.

[98] Smart J.D. (2005). The basics and underlying mechanisms of mucoadhesion. Adv. Drug Deliv. Rev..

[99] Balouiri M., Sadiki M., Ibnsouda S.K. (2016). Methods for in vitro evaluating antimicrobial activity: A review. J. Pharm. Anal..

[100] ICH (2003). Q1A(R2): Stability testing of new drug substances and products. ICH Harmonised Tripartite Guideline Stability.

